# Supplementary figures and images for: ‘More tight-less tight’ Patterns in the Climatic Niche Evolution of Gymnocalycium (Cactaceae): Were Pleistocene Glaciations a Prelude?
Source: PLoS One. 2025 May 20;20(5):e0323758. doi: 10.1371/journal.pone.0323758 (PMC12091827; doi:10.1371/journal.pone.0323758)

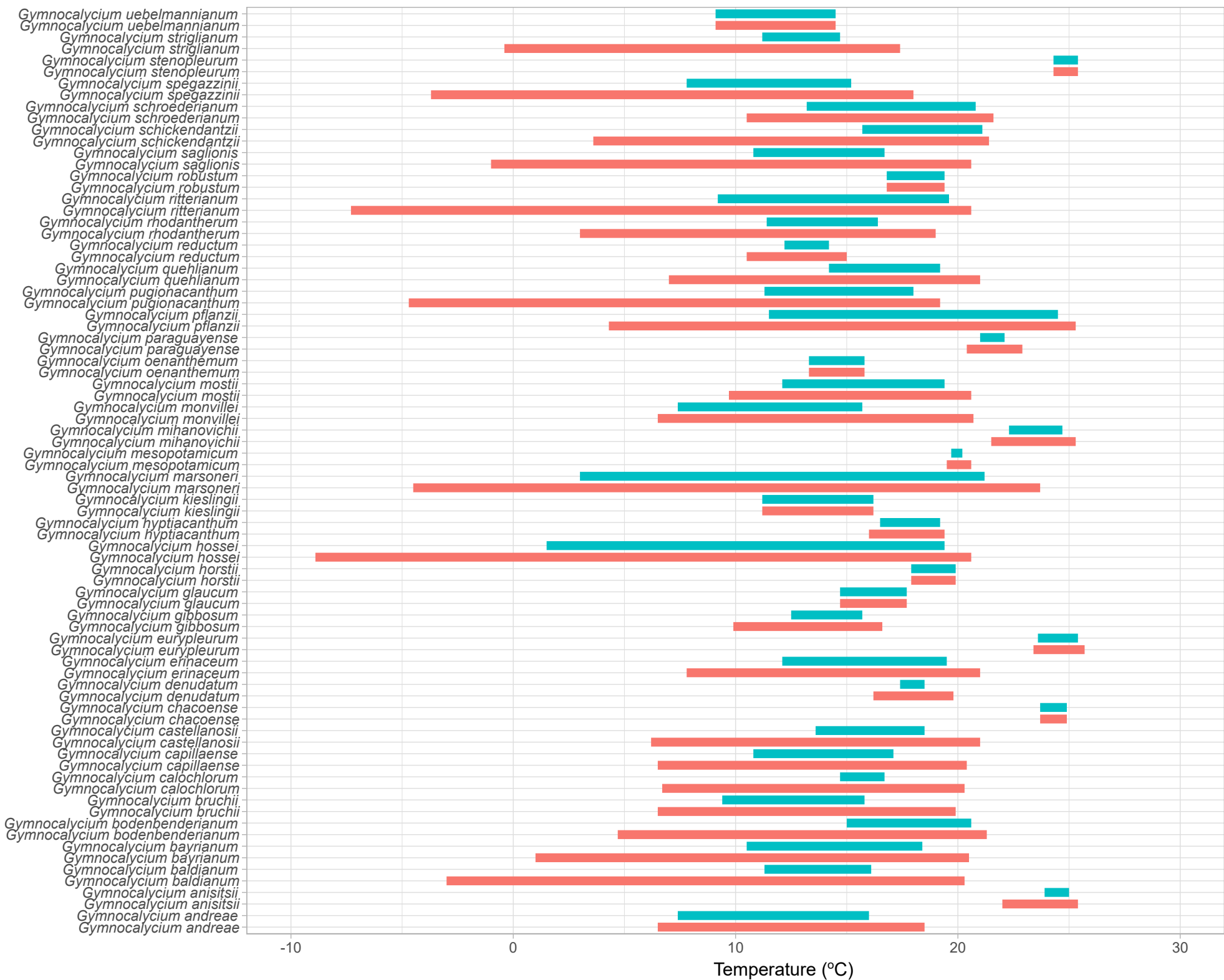

Supplement: S1 Fig — (PDF) [file pone.0323758.s001.pdf]

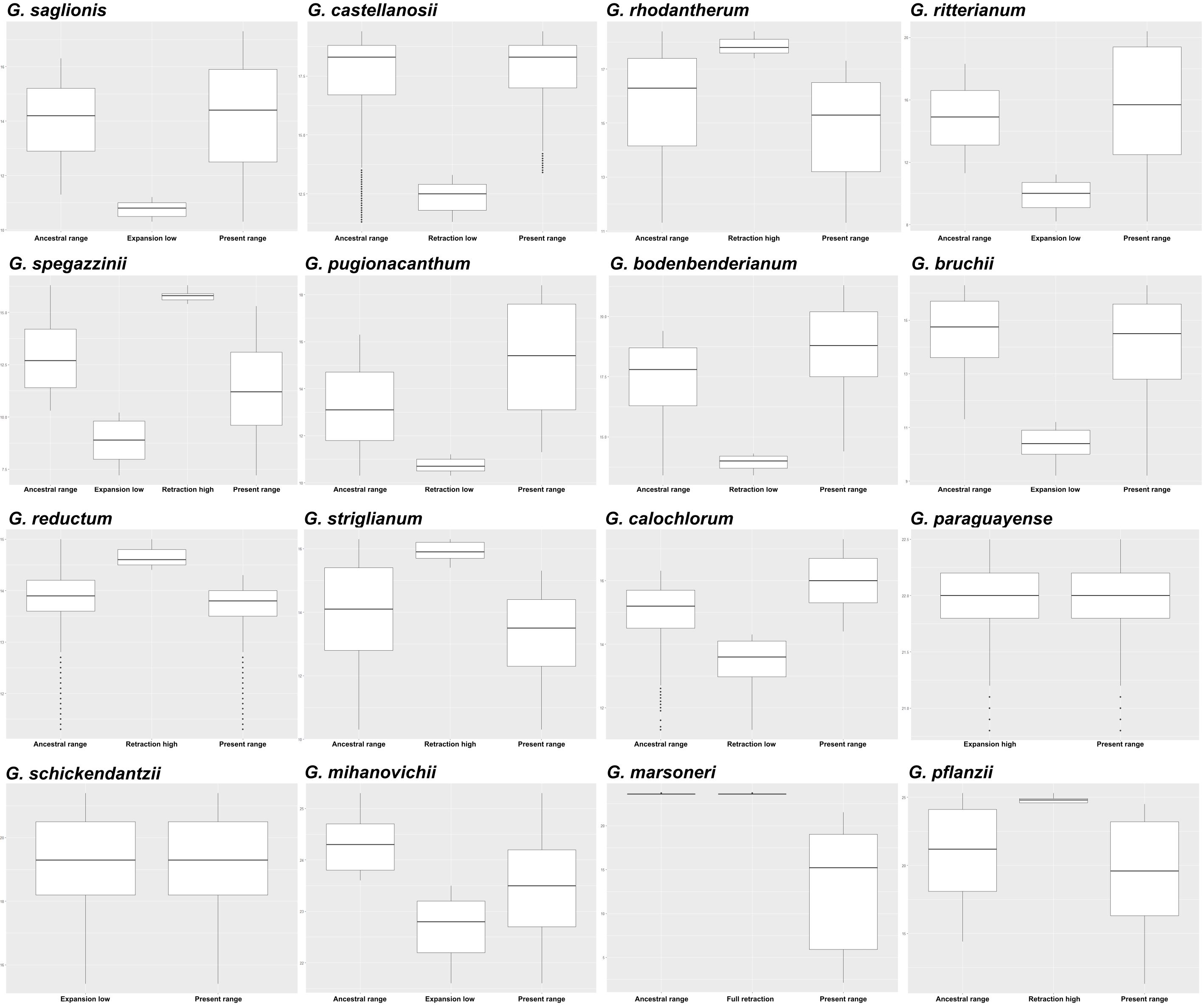

Supplement: S2 Fig — The niche evolutionary events (i.e., retraction and/or expansion) at the upper and/or lower limits with respect to the ancestral niches are represented within the accessible areas (M) of 16 Gymnocalycium species. No ancestral ranges were plotted for G. paraguayense and G. schickendantzii, as their ancestors did not utilize the temperature ranges within their M-areas. No graph was plotted for G. mesopotamicum, as the predicted retraction fell outside the available temperature limits of its M-area. (PNG) [file pone.0323758.s002.png]

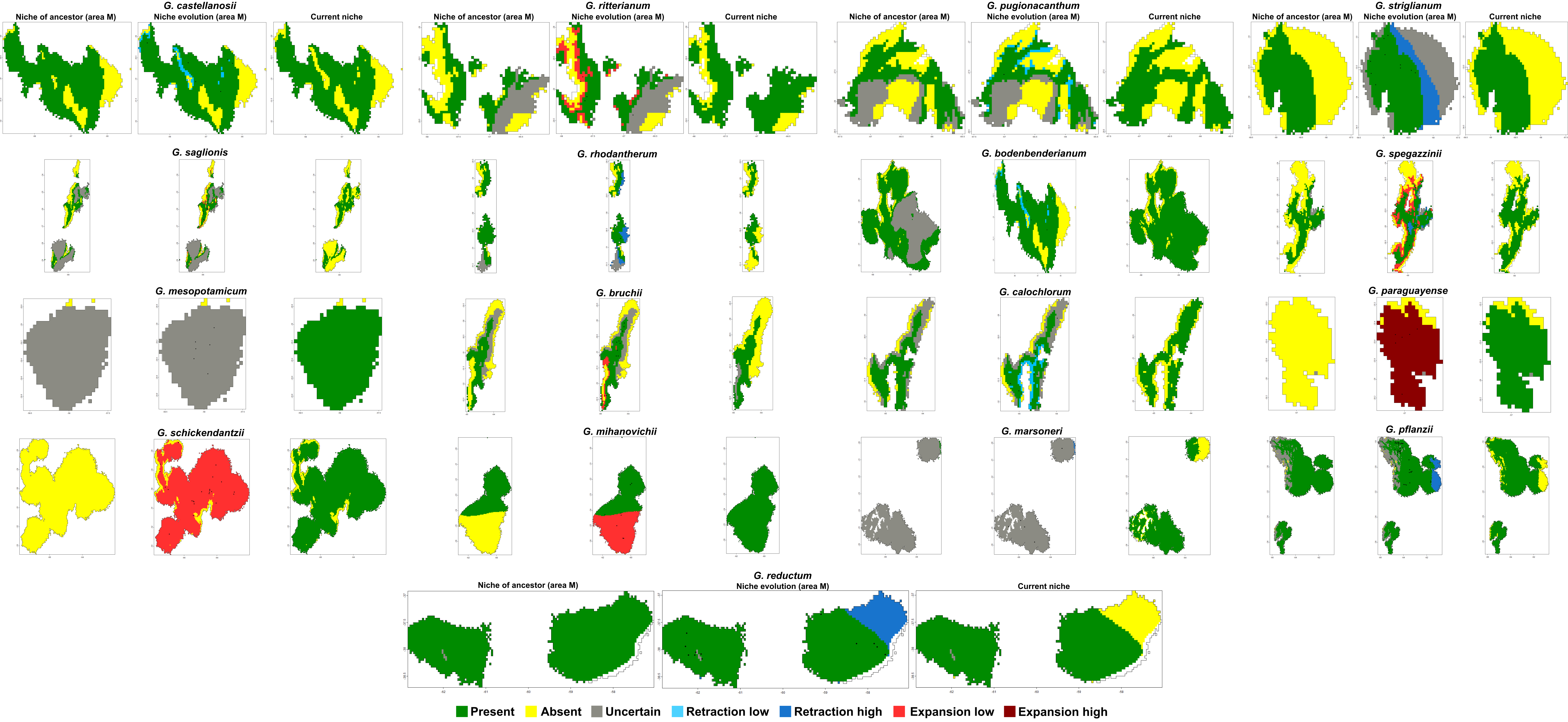

Supplement: S3 Fig — The predicted niche evolutionary events (i.e., retraction and/or expansion) are represented in geographic space. For each species, the ancestral niche, evolutionary changes, and present niche are depicted geographically within the accessible areas (M). (PNG) [file pone.0323758.s003.png]

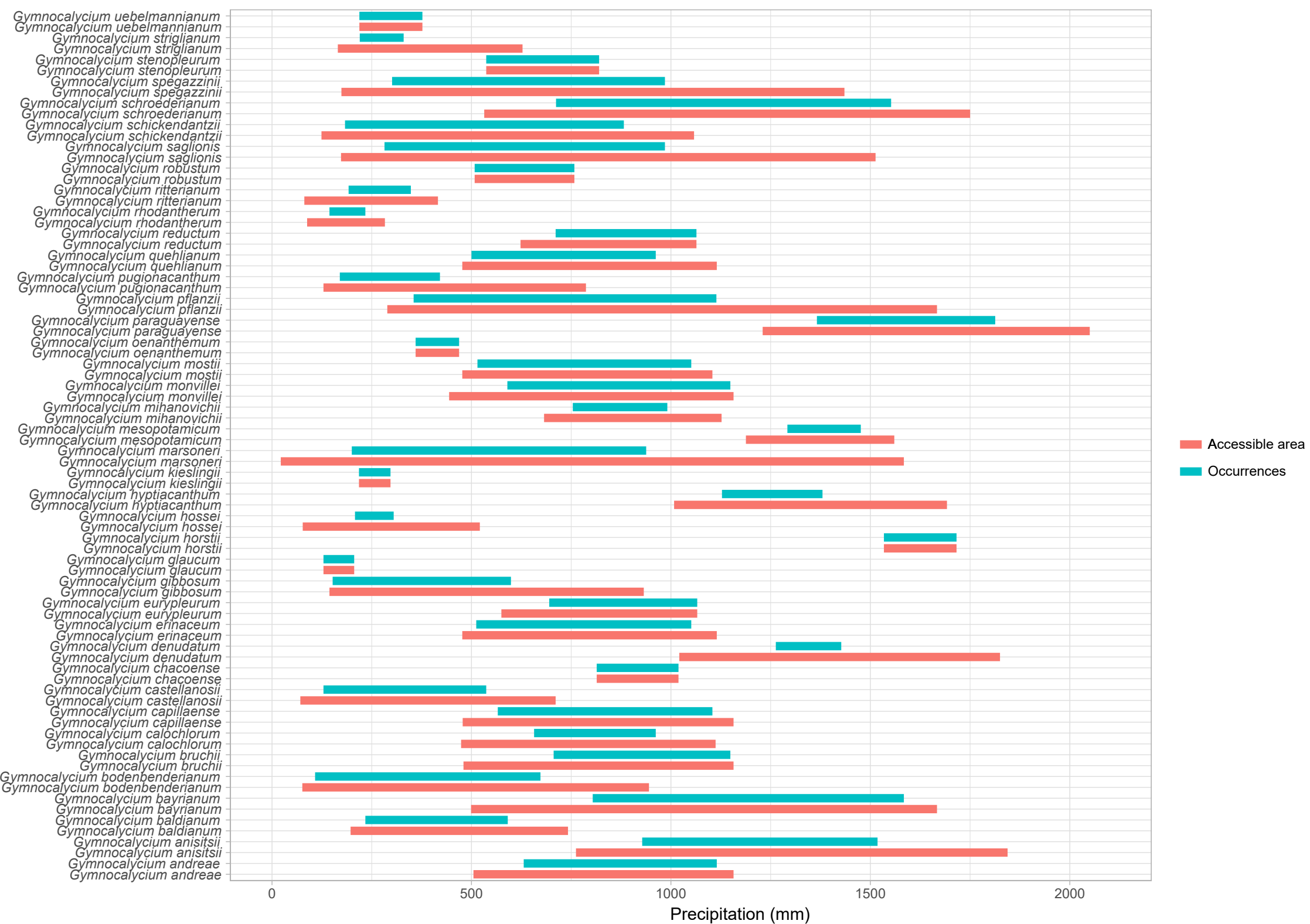

Supplement: S4 Fig — Precipitation ranges based on the accessible areas represent available conditions, and those based on occurrence records represent ranges occupied by the species. (PDF) [file pone.0323758.s004.pdf]

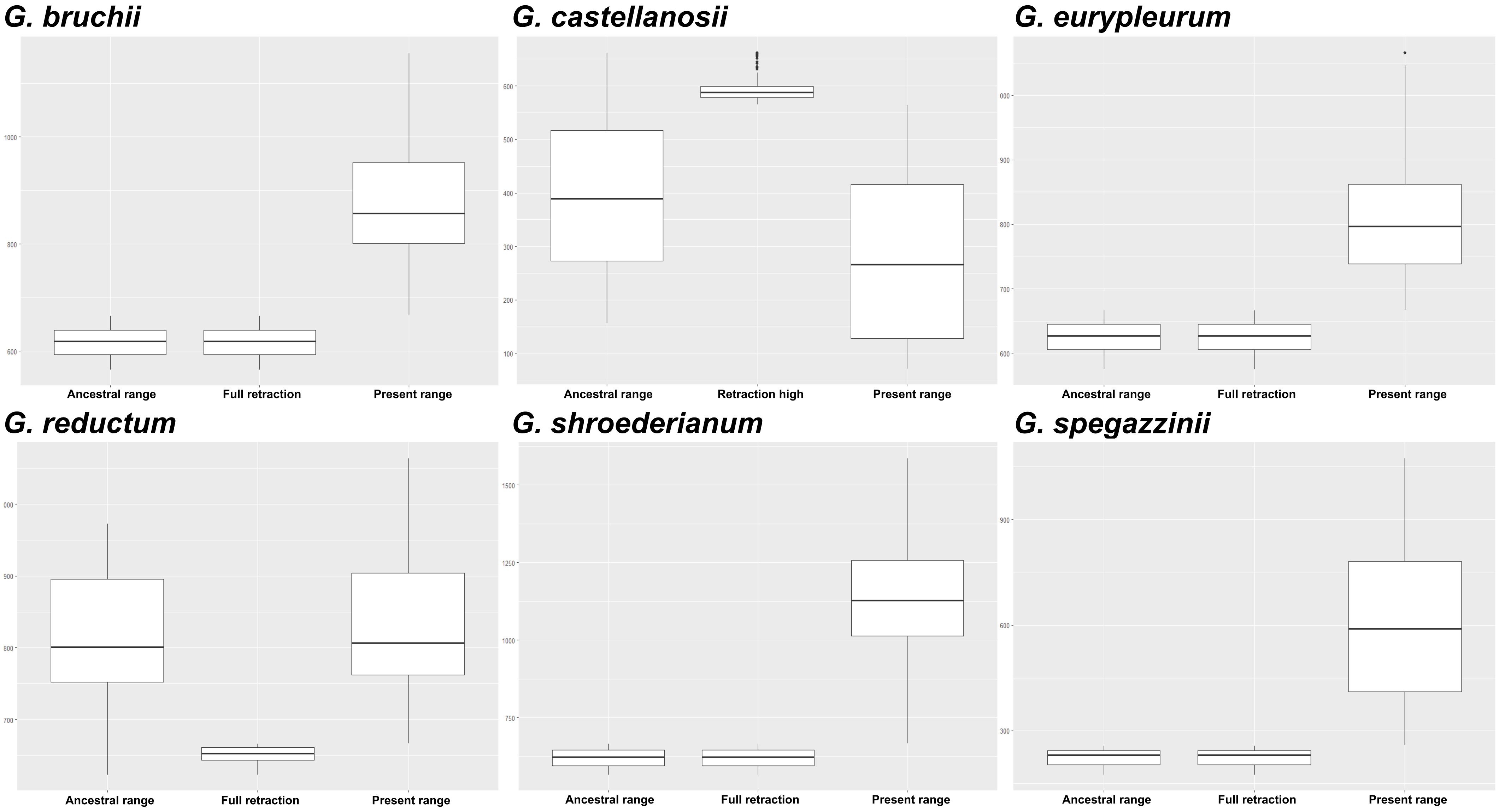

Supplement: S5 Fig — The niche evolutionary events (i.e., retraction and/or expansion) at the upper and/or lower limits with respect to the ancestral niches are represented within the accessible areas (M) of six Gymnocalycium species. No graph was plotted for G. mesopotamicum, as the predicted retraction fell outside the available precipitation limits of its M-area. (PNG) [file pone.0323758.s005.png]

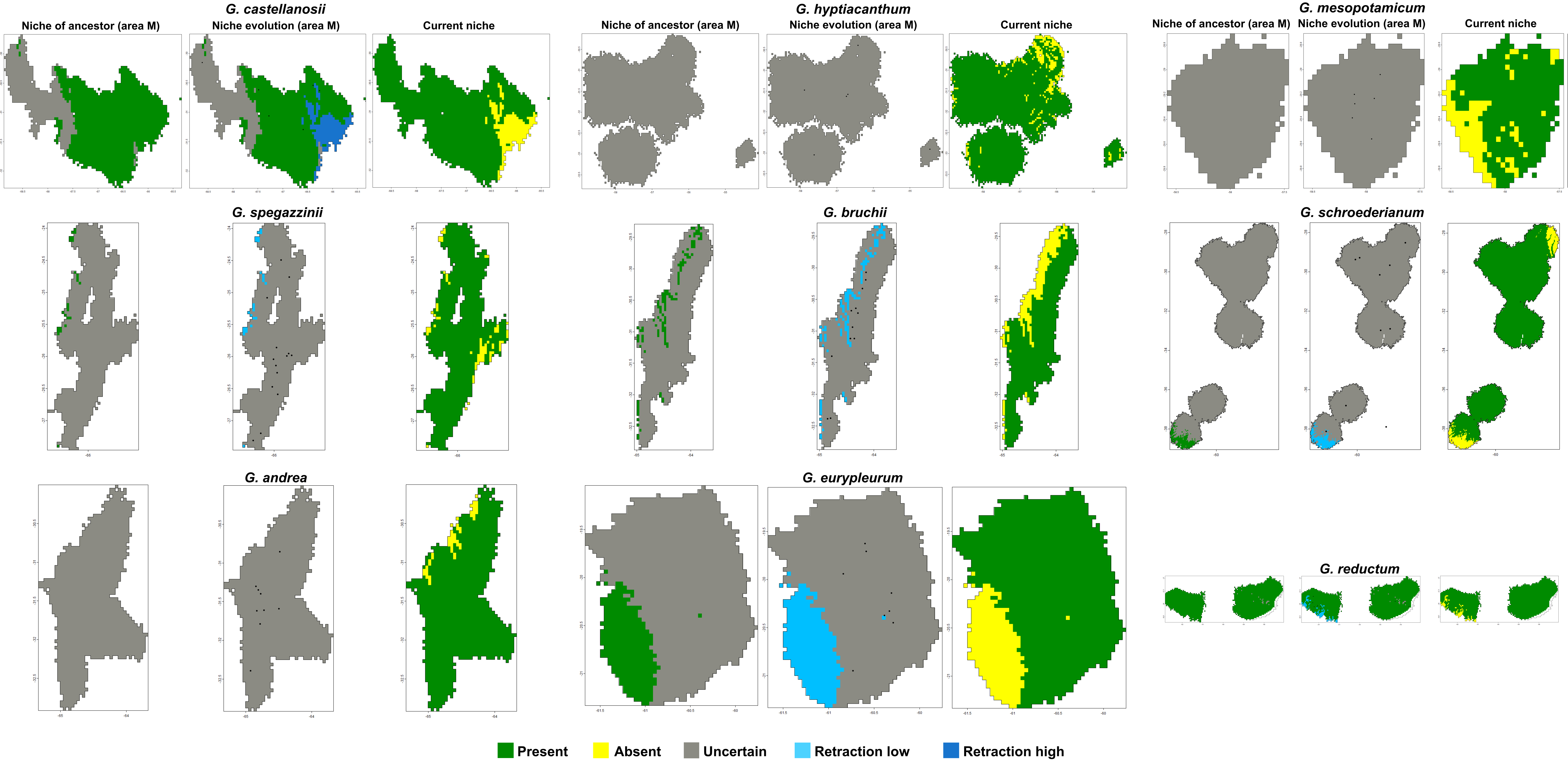

Supplement: S6 Fig — The predicted niche evolutionary events (i.e., retraction and/or expansion) are represented in geographic space. For each species, the ancestral niche, evolutionary changes, and present niche are depicted geographically within the accessible areas (M). (PNG) [file pone.0323758.s006.png]

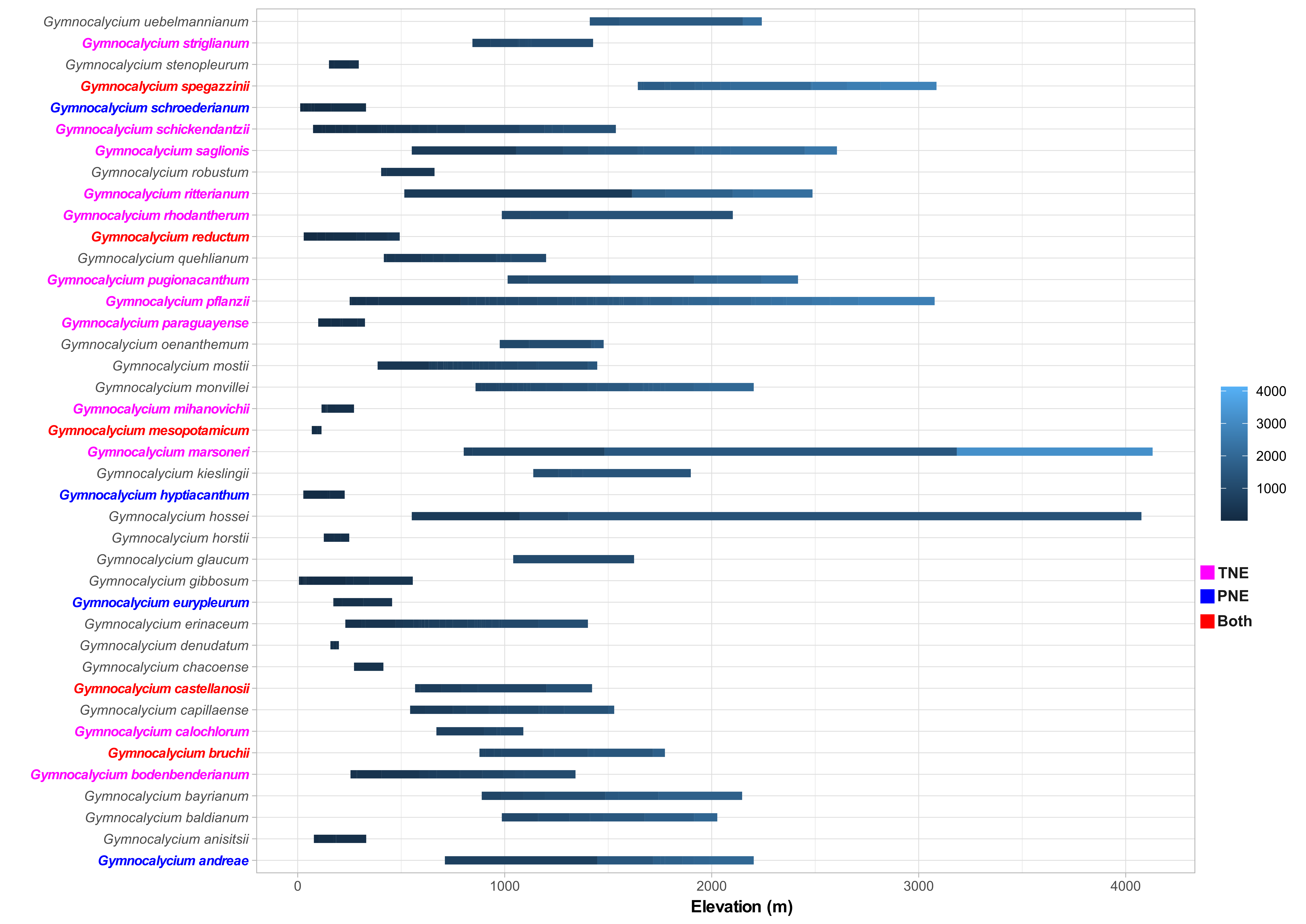

Supplement: S7 Fig — The distribution of the Gymnocalycium species across the altitudinal gradient. The colors indicate the evolutionary specificities with respect to the temperature and precipitation dimensions. TNE - Temperature niche evolution, PNE - Precipitation niche evolution, and Both - Niche evolution in both dimensions. (PNG) [file pone.0323758.s007.png]

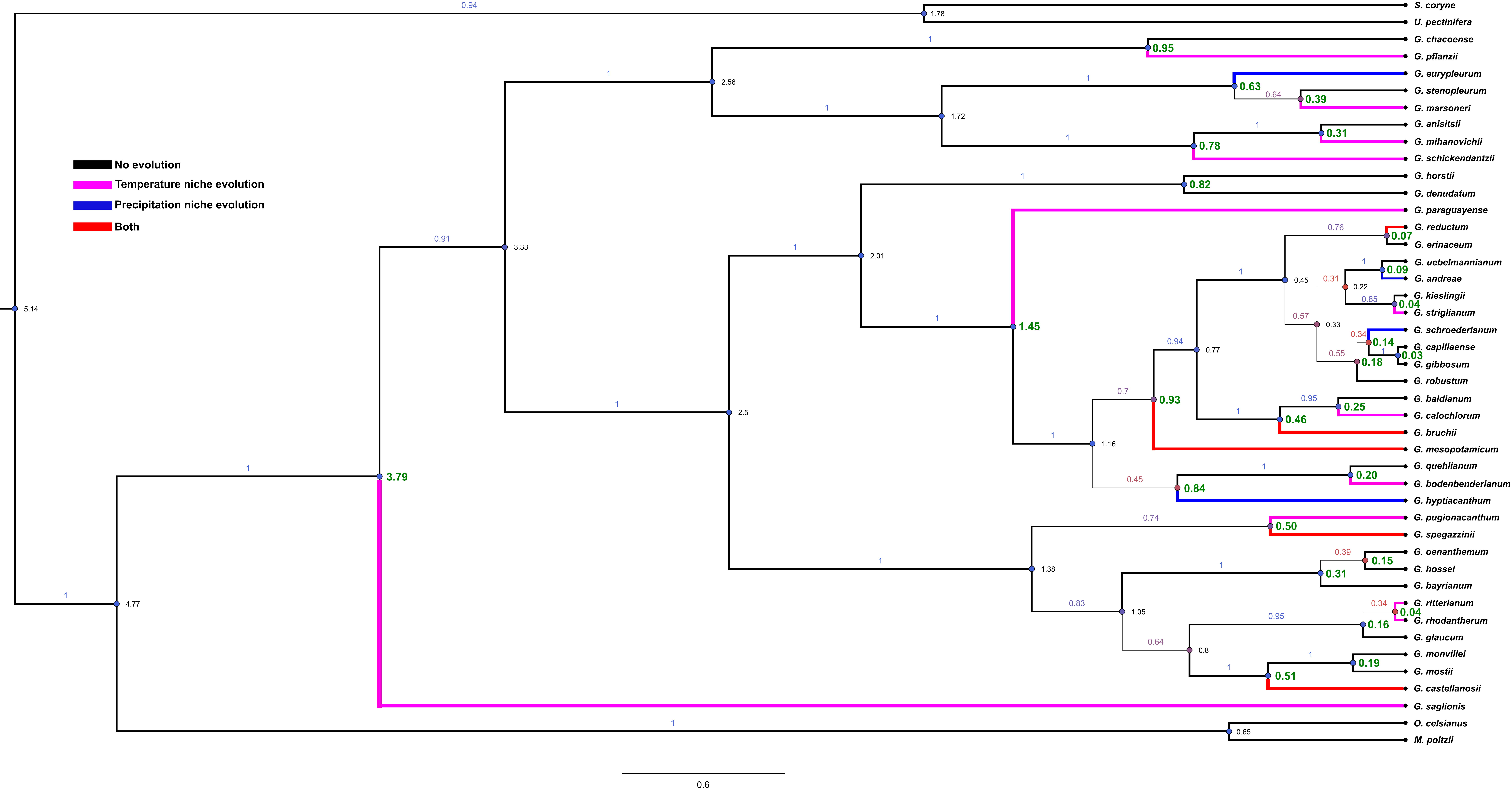

Supplement: S8 Fig — The Bayesian evolutionary tree representing the relationship between the Gymnocalycium species, and their divergence time estimates. The green numerals indicate the divergence time estimates of Gymnocalycium species. The branch colors represent the evolutionary specificities with respect to the temperature and precipitation dimensions. Divergence time estimates are in millions of years. (PNG) [file pone.0323758.s008.png]
