# Supplementary material for: ‘More tight-less tight’ Patterns in the Climatic Niche Evolution of Gymnocalycium (Cactaceae): Were Pleistocene Glaciations a Prelude?
Source: PLoS One. 2025 May 20;20(5):e0323758. doi: 10.1371/journal.pone.0323758 (PMC12091827; doi:10.1371/journal.pone.0323758)
Supplement: S2 Table — Parameterization of the M simulations in the grinnell R package. Simulations were performed for the current scenario (1979–2013). (DOCX) [file pone.0323758.s010.docx]

| **Species** | **Climatic layers** | **Dispersal kernel** | **Dispersal events** | **Kernel spread** | **NdMax*** | **Replications** |
| --- | --- | --- | --- | --- | --- | --- |
| *G. andreae* | PaleoClim 2.5 arcminute | log-normal | 35 | 0.5 | 2 | 10 |
| *G. anisitsii* | PaleoClim 2.5 arcminute | log-normal | 35 | 0.5 | 2 | 10 |
| *G. baldianum* | PaleoClim 2.5 arcminute | log-normal | 35 | 0.5 | 2 | 10 |
| *G. bayrianum* | PaleoClim 2.5 arcminute | log-normal | 35 | 0.5 | 2 | 10 |
| *G. bodenbenderianum* | PaleoClim 2.5 arcminute | log-normal | 35 | 0.5 | 2 | 10 |
| *G. bruchii* | PaleoClim 2.5 arcminute | log-normal | 35 | 0.5 | 2 | 10 |
| *G. calochlorum* | PaleoClim 2.5 arcminute | log-normal | 35 | 0.5 | 2 | 10 |
| *G. capillaense* | PaleoClim 2.5 arcminute | log-normal | 35 | 0.5 | 2 | 10 |
| *G. castellanosii* | PaleoClim 2.5 arcminute | log-normal | 35 | 0.5 | 2 | 10 |
| *G. chacoense* | PaleoClim 2.5 arcminute | log-normal | 35 | 0.5 | 2 | 10 |
| *G. denudatum* | PaleoClim 2.5 arcminute | log-normal | 35 | 0.5 | 2 | 10 |
| *G. erinaceum* | PaleoClim 2.5 arcminute | log-normal | 35 | 0.5 | 2 | 10 |
| *G. eurypleurum* | PaleoClim 2.5 arcminute | log-normal | 35 | 0.5 | 2 | 10 |
| *G. gibbosum* | PaleoClim 2.5 arcminute | log-normal | 35 | 0.5 | 2 | 10 |
| *G. glaucum* | PaleoClim 2.5 arcminute | log-normal | 35 | 0.5 | 2 | 10 |
| *G. horstii* | PaleoClim 2.5 arcminute | log-normal | 35 | 0.5 | 2 | 10 |
| *G. hossei* | PaleoClim 2.5 arcminute | log-normal | 35 | 0.5 | 2 | 10 |
| *G. hyptiacanthum* | PaleoClim 2.5 arcminute | log-normal | 35 | 0.5 | 2 | 10 |
| *G. kieslingii* | PaleoClim 2.5 arcminute | log-normal | 35 | 0.5 | 2 | 10 |
| *G. marsoneri* | PaleoClim 2.5 arcminute | log-normal | 35 | 0.5 | 2 | 10 |
| *G. mesopotamicum* | PaleoClim 2.5 arcminute | log-normal | 35 | 0.5 | 2 | 10 |
| *G. mihanovichii* | PaleoClim 2.5 arcminute | log-normal | 35 | 0.5 | 2 | 10 |
| *G. monvillei* | PaleoClim 2.5 arcminute | log-normal | 35 | 0.5 | 2 | 10 |
| *G. mostii* | PaleoClim 2.5 arcminute | log-normal | 35 | 0.5 | 2 | 10 |
| *G. oenanthemum* | PaleoClim 2.5 arcminute | log-normal | 35 | 0.5 | 2 | 10 |
| *G. paraguayense* | PaleoClim 2.5 arcminute | log-normal | 35 | 0.5 | 2 | 10 |
| *G. pflanzii* | PaleoClim 2.5 arcminute | log-normal | 35 | 0.5 | 2 | 10 |
| *G. pugionacanthum* | PaleoClim 2.5 arcminute | log-normal | 35 | 0.5 | 2 | 10 |
| *G. quehlianum* | PaleoClim 2.5 arcminute | log-normal | 35 | 0.5 | 2 | 10 |
| *G. reductum* | PaleoClim 2.5 arcminute | log-normal | 35 | 0.5 | 2 | 10 |
| *G. rhodantherum* | PaleoClim 2.5 arcminute | log-normal | 35 | 0.5 | 2 | 10 |
| *G. ritterianum* | PaleoClim 2.5 arcminute | log-normal | 35 | 0.5 | 2 | 10 |
| *G. robustum* | PaleoClim 2.5 arcminute | log-normal | 35 | 0.5 | 2 | 10 |
| *G. saglionis* | PaleoClim 2.5 arcminute | log-normal | 35 | 0.5 | 2 | 10 |
| *G. schickendantzii* | PaleoClim 2.5 arcminute | log-normal | 35 | 0.5 | 2 | 10 |
| *G. schroederianum* | PaleoClim 2.5 arcminute | log-normal | 35 | 0.5 | 2 | 10 |
| *G. spegazzinii* | PaleoClim 2.5 arcminute | log-normal | 35 | 0.5 | 2 | 10 |
| *G. stenopleurum* | PaleoClim 2.5 arcminute | log-normal | 35 | 0.5 | 2 | 10 |
| *G. striglianum* | PaleoClim 2.5 arcminute | log-normal | 35 | 0.5 | 2 | 10 |
| *G. uebelmannianum* | PaleoClim 2.5 arcminute | log-normal | 35 | 0.5 | 2 | 10 |

^*NdMax = Maximum number of dispersers^
