# Supplementary material for: ‘More tight-less tight’ Patterns in the Climatic Niche Evolution of Gymnocalycium (Cactaceae): Were Pleistocene Glaciations a Prelude?
Source: PLoS One. 2025 May 20;20(5):e0323758. doi: 10.1371/journal.pone.0323758 (PMC12091827; doi:10.1371/journal.pone.0323758)
Supplement: Attachment — Submitted filename: Responses to reviewers.docx [file pone.0323758.s013.docx]

**From**

Rahul Raveendran Nair

Postdoctoral Researcher

IMBIV, CONICET

Argentina

**To**

Dr. Patrick R. Stephens

Academic Editor

PLOS ONE

Sub: Submission of revised MS (PONE-D-24-56930; ‘More tight-less tight’ Patterns in the Climatic Niche Evolution of *Gymnocalycium* (Cactaceae): Were Pleistocene Glaciations a Prelude?

**Dear Patrick**:

First of all, we would like to thank you very much for allowing us to revise the manuscript based on the reviewers’ suggestions. It is a pleasure to learn that our manuscript could be a nice contribution to the current understanding about the evolutionary dynamics of cacti.

We really value the time and effort that the editor and the reviewers have taken to evaluate the manuscript. We have addressed all comments and suggestions meticulously. One of the major concerns was the choice of the variables. To clarify, we have provided a detailed justification for the selection of variables to the reviewers. We would like to mention the same here also for your kind perusal.

*We understand the significance of choosing non-correlated (independent) variables via either PCA, or variable-elimination through correlation analyses, or through methods such as VIF etc., in conventional ecological niche modeling practices.*

*However, our focus here was different. Our attempt was to reconstruct the ancestral thermal and precipitation niches of Gymnocalycium species using the variables that most represent overall climatic distribution. Hence, we chose only Bio1 variable for the temperature dimension, and Bio12 variable for the precipitation dimension, to perform the analyses. This was not a random selection, and this decision was made during the conceptualization stage of the study.* *Because, these are the two important variables that reflect the overall climatic distribution of a species (Lin et al. 2017; Castro-Insua et al. 2018). The Bio1 and Bio12 are considered to be the “standard climatic variables” (Lin et al. 2017) for studying the overall climatic distribution of a species, and hence, researchers have directly used these variables to study the climatic niche evolution.*

*To avoid the possible confusion, we would like to mention here that we downloaded the full suite of climatic variables from PaleoClim database for employing them in simulating accessible areas (Area* ***M****) only, as an input for grinnell R package.*

*As the BAR method allows the characterization of niches in single dimension at a time, we preferred to study the niche conservatism/evolution using these two important variables (Bio1 and Bio12), that in general, reflect the critical aspects of the species’ overall climatic distribution (Castro-Insua et al. 2018; Wu et al. 2023).*

1. *Castro-Insua, A., Gómez-Rodríguez, C., Wiens, J.J. et al. Climatic niche divergence drives patterns of diversification and richness among mammal families. Sci Rep 8, 8781 (2018).* [*https://doi.org/10.1038/s41598-018-27068-y*](https://doi.org/10.1038/s41598-018-27068-y)
2. *Lin, L.H, and Wiens J.J. Comparing macroecological patterns across continents: evolution of climatic niche breadth in varanid lizards. Ecography 40.8 (2017): 960-970.*
3. *Wu, G.L., Chen, D.X., Zhou, Z., Ye, Q., Baselga, A., Liu, H., Wen, Y. and Nong, S.Q., 2023. Climatic niche divergence explains angiosperm diversification across clades in China. Journal of Systematics and Evolution, 61(4), pp.698-708.*
4. *Owens, H.L., Ribeiro, V., Saupe, E.E., Cobos, M.E., Hosner, P.A., Cooper, J.C., Samy, A.M., Barve, V., Barve, N., Muñoz‐R, C.J. and Peterson, A.T., 2020. Acknowledging uncertainty in evolutionary reconstructions of ecological niches. Ecology and Evolution, 10(14), pp.6967-6977.*

We *have incorporated an additional sentence to justify the selection, as follows:*

Lines 281-283: “*We selected bio1 and bio12, as these variables reflect the most important aspects of the overall climatic distribution of a species [58,99]*”

All other comments were properly addressed and the responses were written separately for the reviewers (appended below). We believe that the manuscript is now improved considerably.

Once again, we would like to express our gratitude to you for spending your valuable time and effort on improving the manuscript.

Thanks

-Rahul

**Responses to the reviewers’ comments**

**Reviewer #1:**

- The study aims to reconstruct ancestral niches for species of the genus *Gymnocalycium*, focusing on abiotic factors such as temperature and precipitation. The research is well-founded and employs a robust methodology that effectively addresses the requirements for hypothesis testing. Overall, this work makes a valuable contribution to understanding the evolutionary dynamics of cacti and is well-suited for publication.

- *We greatly appreciate the time and effort that the reviewer has taken to review this manuscript. We are grateful to the reviewer for sharing positive comments about the quality of our MS. It’s a great pleasure to learn the reviewer’s assessment that our work contributes significantly to the present understanding of the evolutionary dynamics of cacti.*
- Line 75: I would not classify this genus as having a narrow distribution. This characterization conflicts with the statements on lines 346–347. Please revise for consistency.
  - Thanks for the suggestion. As the reviewer suggested, we have modified the sentence as follows:

Lines 76-80:

*“This genus is of particular interest for studying environmental niche evolution across phylogeny due to its (1) high level of endemism [29,31,32], (2) adaptation to heterogenous environmental conditions [29,33–36], and (3) presence at different ecological regions across a range of altitudinal gradients [21,29–32,37,38]”*

- 2. Lines 90–109: Although this critique pertains to style, I found the hypotheses and their justifications difficult to follow (I had to reread a couple of times). I recommend summarizing the hypotheses into two or three key points for clarity and focus. Additionally, consider restructuring the paragraph to present the hypotheses first, followed by their justifications, as this would improve readability.
- *We thank the reviewer for this valuable observation. As suggested, we have modified the said section as follows:*

Lines 81-161:

*In this study, we tested the evolutionary dynamics of climatic niches across the Gymnocalycium phylogeny, asking the following questions.*

*1.	Are the climatic-niches of narrow-ranged Gymnocalycium species highly conserved across the phylogeny, indicating niche conservatism?*

*Various well-established hypotheses, such as niche breadth-range size [39,40], range size-niche breadth [41–43], and range shift-niche breadth [44], that connect niche breadth with the size of geographic ranges, suggest a positive association between niche breadth and geographic range size [45–49]. In a phylogenetic framework, studies on climatic-niche evolution linked with range size suggest that the rate of niche evolution in narrow-ranged species may be lower (i.e., more conserved), compared to wide-ranged species [11,50–52]. However, inter-specific variations in the utilization of environmental space from the total environmental space of the clade may result in the divergence of niches [50]. There are inter-specific differences in the utilization of environmental conditions by the Gymnocalycium species. Hence, we expect that the climatic-niches of narrow-ranged Gymnocalycium species may not be highly conserved across the phylogeny.*

*2.	Do the evolutionary dynamics of thermal and precipitation niches across the Gymnocalycium phylogeny follow similar patterns?*

*As there are strong selective pressures of temperature and precipitation variables on Gymnocalycium species distribution [29,30,33,37,38,53], we expect that the evolutionary dynamics of thermal and precipitation niches across the Gymnocalycium phylogeny do not follow similar patterns.*

*3.	Does a bioregion-specific pattern of niche evolution exist with respect to temperature and precipitation dimensions?*

*Temperature and precipitation variables are central in biogeographic regionalization [54], and the Neotropics have diverse biogeographic regions (bioregions) [55,56]. Hence, we expect a bioregion-specific pattern of niche evolution in Gymnocalycium.*

*4.	Did the Early-Middle Pleistocene glaciations (i.e. GPG and three Post-GPG phases) potentially drive the patterns of lineage divergence in Gymnocalycium species, resulting in the evolution of temperature and precipitation niches?*

*Several studies have linked the evolution of climatic niches with lineage divergence events [14,16,57–61]. In South America, the Great Patagonian Glaciation (GPG; 1.0-1.1 Mya) followed by three Middle Pleistocene glaciations (Post-GPG 1, 2 & 3) [62,63] greatly influenced species diversifications and distributions [64–67], as the glacial cycles of advancement and retreat had significant effects on the climate in southern South America (the regions south of 15^o^S) [68]. Hence, we expect that the Early-Middle Pleistocene glaciations (i.e. GPG and three Post-GPG phases) played a significant role in lineage divergence in Gymnocalycium species, leading to the evolution of temperature and precipitation niches.*

- 3. Lines 141–144: While I agree with the relevance of the variables selected, I am curious why other bioclimatic variables were not included, or why uncorrelated variables were not retained after a PCA of all 19 bioclimatic variables available at Bioclim. For instance, seasonality has been identified as a key driver of Neotropical regionalization, yet Bio15 (precipitation seasonality) was omitted. Please provide additional justification for this selection.
  - *We clearly understand the reviewer’s concern.*

*We understand the significance of choosing non-correlated (independent) variables via either PCA, or variable-elimination through correlation analyses, or through methods such as VIF etc., in conventional ecological niche modeling practices.*

*However, our focus here was different. Our attempt was to reconstruct the ancestral thermal and precipitation niches of Gymnocalycium species using the variables that most represent overall climatic distribution. Hence, we chose only Bio1 variable for the temperature dimension, and Bio12 variable for the precipitation dimension, to perform the analyses. This was not a random selection, and this decision was made during the conceptualization stage of the study.* *Because, these are the two important variables that reflect the overall climatic distribution of a species (Lin et al. 2017; Castro-Insua et al. 2018). The Bio1 and Bio12 are considered to be the “standard climatic variables” (Lin et al. 2017) for studying the overall climatic distribution of a species, and hence, researchers have directly used these variables to study the climatic niche evolution.*

*To avoid the possible confusion, we would like to mention here that we downloaded the full suite of climatic variables from PaleoClim database for employing them in simulating accessible areas (Area* ***M****) only, as an input for grinnell R package.*

*As the BAR method allows the characterization of niches in single dimension at a time, we preferred to study the niche conservatism/evolution using these two important variables (Bio1 and Bio12), that in general, reflect the critical aspects of the species’ overall climatic distribution (Castro-Insua et al. 2018; Wu et al. 2023).*

*Castro-Insua, A., Gómez-Rodríguez, C., Wiens, J.J. et al. Climatic niche divergence drives patterns of diversification and richness among mammal families. Sci Rep 8, 8781 (2018).* [*https://doi.org/10.1038/s41598-018-27068-y*](https://doi.org/10.1038/s41598-018-27068-y)

*Lin, L.H, and Wiens J.J. Comparing macroecological patterns across continents: evolution of climatic niche breadth in varanid lizards. Ecography 40.8 (2017): 960-970.*

*Wu, G.L., Chen, D.X., Zhou, Z., Ye, Q., Baselga, A., Liu, H., Wen, Y. and Nong, S.Q., 2023. Climatic niche divergence explains angiosperm diversification across clades in China. Journal of Systematics and Evolution, 61(4), pp.698-708.*

*Owens, H.L., Ribeiro, V., Saupe, E.E., Cobos, M.E., Hosner, P.A., Cooper, J.C., Samy, A.M., Barve, V., Barve, N., Muñoz‐R, C.J. and Peterson, A.T., 2020. Acknowledging uncertainty in evolutionary reconstructions of ecological niches. Ecology and Evolution, 10(14), pp.6967-6977.*

*As the reviewer suggested, we have incorporated an additional sentence to justify the selection, as follows:*

Lines 281-283:

“*We selected bio1 and bio12, as these variables reflect the most important aspects of the overall climatic distribution of a species [58,99]*”

- 4. Line 194: While the relaxed clock model is a reasonable choice, it might be insightful to test whether a birth–death tree prior would better fit this diverse genus with many narrow endemics. The choice of tree prior significantly influences phylogenetic reconstructions and, consequently, ancestral character reconstructions (in this case: niche). I recommend evaluating model fit by comparing the Birth-Death with alternatives like the Yule model, using tools such as path sampling or stepping-stone sampling in BEAST2 (see: https://www.beast2.org/path-sampling/). This approach would enhance the robustness of age estimates and lend greater confidence to the hypotheses tested.
- *Thanks for the suggestion. We really appreciate the reviewers’ effort to suggest other possibilities.*

*We chose Yule tree prior as our dataset involves DNA sequences of different species within the Gymnocalycium genus. This tree prior has been widely recommended to study speciation processes. If the sequence data is sufficiently informative, it has been recently reported that the choice of tree prior and molecular clock does not significantly affect the phylogenetic divergence estimates (Sarver et al. 2019).*

*Sarver BAJ, Pennell MW, Brown JW, Keeble S, Hardwick KM, Sullivan J, Harmon LJ. 2019. The choice of tree prior and molecular clock does not substantially affect phylogenetic inferences of diversification rates. PeerJ 7:e6334* [*https://doi.org/10.7717/peerj.6334*](https://doi.org/10.7717/peerj.6334)

*We believe that our sequence data is sufficiently informative, as the same molecular data was used earlier to study the species relationship within the Gymnocalycium genus (Demaio et al. 2011).*

*Demaio, P. H., Barfuss, M. H., Kiesling, R., Till, W., & Chiapella, J. O. (2011). Molecular phylogeny of Gymnocalycium (Cactaceae): assessment of alternative infrageneric systems, a new subgenus, and trends in the evolution of the genus. American Journal of Botany, 98(11), 1841-1854.*

*The first author of the above-publication (Pablo H. Demaio) is a co-author of this manuscript.*

*Hence, we prefer to move forward with the Yule tree prior in this manuscript.*

5. Figures: The manuscript includes 10 figures, which may overwhelm readers. I suggest transferring at least six figures to the supplementary material or consolidating them into more synthetic, comprehensive figures.

- Thanks for this suggestion. We understand the reviewer’s concern. As suggested, six figures (Figures 3, 5, 6, 7, 9, and 10) were shifted to the supplementary section. Only four figures are placed in the main text.

**Reviewer #2:**

- This is my first review of the manuscript entitled, “‘More tight-less tight’ Patterns in the Climatic Niche Evolution of *Gymnocalycium* (Cactaceae): Were Pleistocene Glaciations a Prelude?” This is a well-written and engaging paper in which the authors investigate the factors contributing to the niche evolution of a narrowly distributed cactus genus. Despite its restricted distribution and endemism, the genus appears to be a suitable model for this type of study, as different species occupy a range of heterogeneous environmental conditions. Studies like this are still scarce in the cactus family, and I believe this paper represents a valuable empirical contribution to the field. However, I have a few concerns that I would like the authors to address.
- *We greatly appreciate the time and effort that the reviewer has taken to review this manuscript. We are really thankful to the reviewer for sharing positive observations about the manuscript such that the manuscript could be a valuable empirical contribution to the current understanding of the evolutionary dynamics of cacti.*

Major Comments

- Selection of Variables. My primary concern relates to the selection of variables used to model niche evolution. The argumentation is based solely on results obtained from two variables, and the authors do not mention any tests to justify their selection. This raises questions about whether these variables truly represent the most relevant factors for niche evolution.
  - *We clearly understand the reviewer’s concern.*

*We understand the significance of choosing non-correlated (independent) variables via either PCA, or variable-elimination through correlation analyses, or through methods such as VIF etc., in conventional ecological niche modeling practices.*

*However, our focus here was different. Our attempt was to reconstruct the ancestral thermal and precipitation niches of Gymnocalycium species using the variables that most represent overall climatic distribution. Hence, we chose only Bio1 variable for the temperature dimension, and Bio12 variable for the precipitation dimension, to perform the analyses. This was not a random selection, and this decision was made during the conceptualization stage of the study.* *Because, these are the two important variables that reflect the overall climatic distribution of a species (Lin et al. 2017; Castro-Insua et al. 2018). The Bio1 and Bio12 are considered to be the “standard climatic variables” (Lin et al. 2017) for studying the overall climatic distribution of a species, and hence, researchers have directly used these variables to study the climatic niche evolution.*

*To avoid the possible confusion, we would like to mention here that we downloaded the full suite of climatic variables from PaleoClim database for employing them in simulating accessible areas (Area* ***M****) only, as an input for grinnell R package.*

*As the BAR method allows the characterization of niches in single dimension at a time, we preferred to study the niche conservatism/evolution using these two important variables (Bio1 and Bio12), that in general, reflect the critical aspects of the species’ overall climatic distribution (Castro-Insua et al. 2018; Wu et al. 2023).*

*Castro-Insua, A., Gómez-Rodríguez, C., Wiens, J.J. et al. Climatic niche divergence drives patterns of diversification and richness among mammal families. Sci Rep 8, 8781 (2018).* [*https://doi.org/10.1038/s41598-018-27068-y*](https://doi.org/10.1038/s41598-018-27068-y)

*Lin, L.H, and Wiens J.J. Comparing macroecological patterns across continents: evolution of climatic niche breadth in varanid lizards. Ecography 40.8 (2017): 960-970.*

*Wu, G.L., Chen, D.X., Zhou, Z., Ye, Q., Baselga, A., Liu, H., Wen, Y. and Nong, S.Q., 2023. Climatic niche divergence explains angiosperm diversification across clades in China. Journal of Systematics and Evolution, 61(4), pp.698-708.*

*Owens, H.L., Ribeiro, V., Saupe, E.E., Cobos, M.E., Hosner, P.A., Cooper, J.C., Samy, A.M., Barve, V., Barve, N., Muñoz‐R, C.J. and Peterson, A.T., 2020. Acknowledging uncertainty in evolutionary reconstructions of ecological niches. Ecology and Evolution, 10(14), pp.6967-6977.*

*As the reviewer suggested, we have incorporated an additional sentence to justify the selection, as follows:*

Lines 281-283:

“*We selected bio1 and bio12, as these variables reflect the most important aspects of the overall climatic distribution of a species [58,99]*”

Specific Comments:

- Lines 123-125: Were the analyses also performed without the extrapolated samples? I am concerned about the potential bias these samples could introduce to the model. Please provide more details about how many samples fall into this category.
- *We understand the reviewer’s concerns. Here, extrapolation implies that we sourced geographical coordinates of exact localities that were well documented by collectors/researchers, from the google map. In the dataset, less than 5% of the total records fall into this category. The veracity of such records was meticulously vetted by the field ecologists Diego E. Gurvich and Pable H. Demaio who predominantly work on Cacti in Latin America. Both of them are associated with this manuscript.*
- 2. Line 132: Relying on only two records for *G. chacoense* seems insufficient for robust modeling. Please address this limitation.
- *We understand the reviewer’s concern. As the distributional information of G. chacoense was scarce, we did not make any assumption/hypotheses about the accessible area of this species because* ***M*** *simulation failed for this species. Instead, we sourced the environmental information from the known-occurrences, and assigned the code “uncertain = ?” to the bins that exist beyond the lower and upper limits of the occupied environmental ranges (Supplementary file 2). BAR method enables the user to incorporate uncertainty associated with limits of the climatic distribution of such species. To avoid all possible confusions, we incorporated the following sentence in the subsection ‘Characterization of ancestral niches’ in the methodology.*

Lines 303-306:

“*In the binning scheme of species for which the* ***M*** *simulation failed due to a low number of records, the bins located beyond the lower and upper limits of the occupied environmental ranges were coded as ‘uncertain’ to incorporate the uncertainty associated with climatic distribution of such species*”

- 3. Lines 185-187: Please, provide more details about the molecular data used. Where the sequences could be found?
  - The molecular data is sourced from the publication mentioned below

*Demaio, P. H., Barfuss, M. H., Kiesling, R., Till, W., & Chiapella, J. O. (2011). Molecular phylogeny of Gymnocalycium (Cactaceae): assessment of alternative infrageneric systems, a new subgenus, and trends in the evolution of the genus. American Journal of Botany, 98(11), 1841-1854.*

*The first author of the above-publication (Pablo H. Demaio) is a co-author of this manuscript.*

*The information about these sequences was already provided in the text.*

Lines: 251-255

*“For molecular dating****,*** *we performed a Bayesian phylogenetic reconstruction using the aligned DNA sequence matrix (6195-bp) of four plastid markers viz., atpI-atpH, petL-psbE, trnT-trnL-trnF, and trnK-matK, that was employed earlier for developing the latest available phylogeny of the Gymnocalycium group that display inter-specific relationships [69]”*

BEAST and BEAUTi Usage

4. Lines 200-201: It appears that the analysis was conducted in BEAST, while BEAUTi was used primarily for setting priors. Please clarify this in the manuscript.

- Thanks a lot for bringing this into our attention. We have changed the sentence as follows:

Lines 270-271:

“*The input file was prepared in BEAUti, and the divergence time estimation was performed using BEAST v.1.7.5 [94]*”

Validation of Selected Variables

- 5. Lines 208-211: The manuscript lacks any mention of tests to validate the chosen variables for estimating niche evolution. I recommend conducting a test to confirm the relevance of these variables for the species' current niches in comparison to other available variables.
- *We request the reviewer to kindly check the explanation given above. We have already provided our justification under the ‘selection of variables’ (Section: Major comments).*

Figures and Legends

- 6. Figure 2: Could you associate each projection of accessible areas with a geographic map? It is challenging to identify the occurrence regions for each species in the current format.
- *We understand the concern of the reviewer. The said figure is modified by placing* ***M****s on a larger geographic map.*
- 7. Figure 3 Legend: The terms “utilization” and “utilized” may not be appropriate. Species do not actively “use” temperature ranges; instead, they exhibit ecological requirements that permit them to occupy these ranges. Please revise the language.
- *Figures 3 has now become a supplementary figure. As suggested by the reviewer, legends of two supplementary figures were changed as follows;*

***S1 Fig. Available and occupied temperature ranges:*** *Temperature ranges based on the accessible areas represent available conditions, and those based on occurrence records represent ranges occupied by the species.*

***S4 Fig. Available and occupied precipitation ranges.*** *Precipitation ranges based on the accessible areas represent available conditions, and those based on occurrence records represent ranges occupied by the species.*

- 8. Figure 3: Overlapping the occurrence and accessible area data would make the figure more informative. This would allow for better visualization of how variables in accessible areas correspond to those in occurrence areas.
- Thanks for this suggestion. As suggested, the figures were revised (S1 Fig and S4 Fig).
- 9. Figure 6: When is the expansion considered high or low? I suggest including a discussion of this point in the text.
  - *As suggested by the reviewer, the following sentence is incorporated in the subsection ‘Characterization of ancestral niches’ of the methodology section.*

Lines 315-320:

*“*If the current niche of the descendent species exceeds the upper limit of its ancestral niche, the niche change is termed as ‘expansion high’, and if the current niche range exceeds the lower limit of its ancestral niche, it is ‘expansion low’. Similarly, if the niche of the descendent species retracts from the upper limit of its ancestral niche, it is ‘retraction high’, and if the retraction occurs at the lower limit, it is ‘retraction low’*”*

Minor Comments

- 1. Lines 78-81: The sentence is unclear. Please rephrase for better readability.
  - *As suggested by the reviewer, the sentence is modified as follows:*

Lines 85-87:

“*Various well-established hypotheses, such as niche breadth-range size [39,40], range size-niche breadth [41–43], and range shift-niche breadth [44], that connect niche breadth with the size of geographic ranges, suggest a positive association between niche breadth and geographic range size [45–49]*”

- 2. Line 117: Replace “global biodiversity information facility” with “Global Biodiversity Information Facility (GBIF)” and provide a link to the database.
  - *As suggested by the reviewer, we capitalized each word, and provided a link to the database.*
- 3. Abbreviation Consistency: Please check the abbreviation of the genus name throughout the manuscript for consistency.
  - *It is checked. We used the full name of the genus (Gymnocalycium) in instances where either the genus itself is mentioned, or when the sentence starts with the name of a Gymnocalycium species.*
- 4. Lines 415-417: This sentence is better suited for the introduction. Consider relocating it.
  - *As the reviewer suggested, the sentence is shifted to the introduction (Lines 73-76).*
- 5. Lines 419-420: Where are the results of the dated phylogeny? While I understand this is not the main focus of the paper, a brief discussion of diversification times would add value.
  - *The dated phylogeny is given as a supplementary figure (S8 Fig.). As the reviewer has rightly mentioned, dated phylogeny was not our main focus. But we have mentioned important results related to the diversification times, relevant to our study, in the subsection “Niche changes at the interface of paleoclimatic changes” of the ‘Results’ section [lines 506-546].*
