# Supplementary material for: ‘More tight-less tight’ Patterns in the Climatic Niche Evolution of Gymnocalycium (Cactaceae): Were Pleistocene Glaciations a Prelude?
Source: PLoS One. 2025 May 20;20(5):e0323758. doi: 10.1371/journal.pone.0323758 (PMC12091827; doi:10.1371/journal.pone.0323758)
Supplement: Attachment — Submitted filename: Response to Reviewer.docx [file pone.0323758.s014.docx]

From	04/09/2025

**Rahul Raveendran Nair**

Postdoctoral researcher

IMBIV, CONICET

Argentina

To

**Dr. Patrick R. Stephens**

The Academic Editor

PloS One

Sub: Submission of revised manuscript titled “‘More tight-less tight’ Patterns in the Climatic Niche Evolution of *Gymnocalycium* (Cactaceae): Were Pleistocene Glaciations a Prelude?”, PONE-D-24-56930R1

Dear Patrick:

Many thanks for allowing us to improve our manuscript. As suggested by you and the reviewer, we have made necessary changes in the manuscript. Authors’ responses to the comments offered by the editor and the reviewer (Page no: 3) can be found below.

Editor’s comments:

- First off, my apologies for how long this review process took. One of the original reviewers declined to consider the revision, and it took considerable time to secure a new second reviewer. The new reviewer has a number of suggestions related to clarity, and also suggests some new topics that may be worth addressing. I agree with the reviewer that the Binned ancestral range reconstruction (BAR) method will likely still be unfamiliar to many readers, and so articulating the advantage of this approach, as well as the trade-off it perhaps represents, over the more familiar method of simply using maximum likelihood and ignoring uncertainty would help readers understand why you chose this method. The discussion in Owens et al (2020) might be helpful for this.

Owens, H. L., Ribeiro, V., Saupe, E. E., Cobos, M. E., Hosner, P. A., Cooper, J. C., ... & Peterson, A. T. (2020). Acknowledging uncertainty in evolutionary reconstructions of ecological niches. Ecology and Evolution, 10(14), 6967-6977.

- *We understand the complexities of review process. We thank you for all your effort for shaping the manuscript by letting us to receive constructive feedbacks. As suggested by the reviewer, a paragraph is incorporated in the beginning of discussion detailing the advantages and limitations of the BAR method.*
- "The model appears oversimplified, as the used parameters assumes fixed numbers of events and dispersers across all species. Is this assumption in supported by empirical data for each species? Do all *Gymnocalycium* species have the same dispersal mechanisms and exhibit the same dispersal rates? Please explain these parameters choice and provide an evaluation of how potential variations in dispersal rates might influence the results." It is not entirely clear to me whether the way that you have implemented BAR is as rigid as the reviewer implies. My reading of the method is that it does not require such strict assumptions. However, this comment does indicate either a methodological detail that needs to be better justified or a potential misunderstanding that you should head off in the minds or readers by being clearer about how this unfamiliar method works.
- *We have provided a detailed explanation for the reviewer, and also incorporated additional sentences in the subsection ‘Accessible area hypothesis’ of the section ‘Methods’ to bring clarity.*
- I believe that the keywords you have chosen are appropriate, and I see no issue with them overlapping with the title. However, feel free to retain them, change them or add additional key words as you wish.
- We have replaced three keywords with other relevant words.
- Overall, this should be a fairly minor revision for the sake to clarity. However, the issues raised by reviewer two seem substantial enough to me that I will likely send the revision back out for a third round of review. That said, since one reviewer has already signed on this draft, I will not require two reviews of your revision.
- Many thanks for your efforts. We are grateful to you for having given this opportunity to improve the manuscript.

We believe that we have performed all modifications/edits suggested by the third reviewer. We request you to kindly process our manuscript.

Thanks

Rahul (Corresponding author)

**Responses to the reviewer’s comments**

**Reviewer #3:**

•	I would like to thank the editor for the invitation to review this manuscript. I found it highly interesting and appreciated the opportunity to explore some approaches that were previously unfamiliar to me. I received a revised version of the manuscript ‘More tight-less tight’ Patterns in the Climatic Niche Evolution of *Gymnocalycium* (Cactaceae): Were Pleistocene Glaciations a Prelude?”, which includes tracked changes and a response from the authors to previous reviewer comments. While I did not participate in the initial review round, I have examined both the revised manuscript and the authors' responses. Several of my own questions have already been satisfactorily answered, and overall, the study presents a well-structured investigation into the ecological and evolutionary dynamics of *Gymnocalycium* species.

o	*We wholeheartedly thank the reviewer for having spent considerable time and effort in reviewing our manuscript by considering our previous responses to the reviewers. We appreciate the reviewer’s assessment that the manuscript details a well-structured investigation of the ecological and evolutionary dynamics of Gymnocalycium species.*

•	The authors provide a strong theoretical foundation for their hypotheses and employ the Binned Ancestral Range (BAR) method to reconstruct past climatic niches, allowing for direct comparisons between precipitation and temperature variables. The manuscript applies adequate methodology and contributes valuable insights, but certain aspects require further clarification and refinement. Below, I outline specific points that I encourage the authors to address to improve its scientific rigor and practical implications.

o	*Thanks for all these critical suggestions. We have addressed all your concerns. Necessary changes are incorporated in the manuscript at the appropriate places.*

- Keywords: The keywords repeat terms from the title. I recommend replacing redundant keywords with additional relevant terms to improve the manuscript’s discoverability in databases
  - *Thanks for the suggestion. As the reviewer suggested, three redundant keywords were removed, and replaced with the words that are important in the context of our study. The keyword Gymnocalycium is retained because of its high significance related to the study. The current list of keywords includes Gymnocalycium, niche conservatism, precipitation, temperature, expansion, retraction.*
- Methodology: This was my main concern about the manuscript: the construction of the dispersal model. While the authors employ a novel and sophisticated approach to define the species accessible area hypothesis, the model appears oversimplified, as the used parameters assumes fixed numbers of events and dispersers across all species. Is this assumption in supported by empirical data for each species? Do all *Gymnocalycium* species have the same dispersal mechanisms and exhibit the same dispersal rates? Please explain these parameters choice and provide an evaluation of how potential variations in dispersal rates might influence the results.
- *We have understood the reviewer’s concern.*

*We used a simulation-based estimation of accessible areas for the 40 species of Gymnocalycium. The first version of this simulation was discussed in Barve et al. 2011, and it was based on fixed rules such that the movement is only allowed to neighboring cells. Later in 2021, Machado-Stredel et al. developed an extension to this method by incorporating a probability density function (dispersal kernel) that explains the probability of movement of dispersers to any position relative to the ‘starting population’. Then, the simulation of colonization and extinction events in cell grids occur based on the suitability of environmental conditions, assessed from a preliminary estimate of the fundamental ecological niche. These simulations were performed in several replications. In each replication, a subset of presence points would be kept as the ‘starting population’.*

***Barve, N****., Barve, V., Jiménez-Valverde, A., Lira-Noriega, A., Maher, S.P., Peterson, A.T., Soberón, J. and Villalobos, F., 2011. The crucial role of the accessible area in ecological niche modeling and species distribution modeling. Ecological modelling, 222(11), pp.1810-1819.*

***Machado-Stredel, F****., Cobos, M.E. and Peterson, A.T., 2021. A simulation-based method for selecting calibration areas for ecological niche models and species distribution models. Frontiers of Biogeography, 13(4).*

*The seed dispersal events in Gymnocalycium species are mainly mediated by the ants. As of now, no empirical studies on dispersal events have been reported in this genus so far. The two co-authors Pablo H. Demaio and Diego E. Gurvich have more than two decades of working knowledge in the field exploration of this genus. Their google scholar profiles are given below for the ease of verification, if necessary.*

*PHD:* [*https://scholar.google.com.ar/citations?hl=en&user=fvX88eoAAAAJ*](https://scholar.google.com.ar/citations?hl=en&user=fvX88eoAAAAJ)

*DEG:* [*https://scholar.google.com/citations?user=RUNPpC8AAAAJ&hl=it*](https://scholar.google.com/citations?user=RUNPpC8AAAAJ&hl=it)

*All simulated accessible areas were visually inspected by them to assess the plausibility of the accessibility of the current populations. Maximum dispersal events that can happen in 35 years are set to 35 (one per year) for all species. This fixed number (one per year) was chosen considering the two facts.*

i) *PHD and DEG have a strong conviction from their decades of field explorations that the dispersal events in Gymnocalycium can rarely exceed one in any of these species.*
ii) *As already mentioned above, no empirical studies on dispersal events have been conducted in Gymnocalycium so far.*

*However, despite all the above facts, we believe that empirical investigations on dispersal would be required to determine the accurate number of dispersal events in all these species.*

*We have specifically mentioned in the methodology that;*

*“Finally, the* ***M****s that were simulated with an SD of 0.50 were chosen for further analyses (Fig 2). This selection was performed by superimposing all* ***M*** *polygons on the South American map, and choosing the appropriate* ***M****s based on visual inspection and expert knowledge.” Getting realistic accessible areas that can be accessed by populations over a time span of 35 years was our prime interest.*

*From each colonized cell, dispersers’ movement per dispersal event will be based on the suitability of the cell. The suitability range (0-1) will be divided into ‘n’ number of intervals based on the maximum number of disperses; in this case, it is two. It indicates that per dispersal event, if the suitability of a cell (s) is < 0.5, only one disperser will move from that cell, and if the value of s > 0.5, two dispersers will move from that cell. Considering the present geographic distribution, and low levels of dispersal events per year, we chose to provide a platform where two levels of suitability (less suitable, s < 0.5 and more suitable, s > 0.5) were considered in each dispersal event from a colonized cell, and hence, the maximum number of dispersers was set to two.*

*For the ease of understanding, the following sentence is added under the sub-section ‘Accessible area hypothesis’ (Lines 187 – 192).*

*“The parameter (maximum number of dispersers) splits the suitability range (0-1) of a colonized cell into a number of intervals equal to the value of the parameter [84]. In this case, the suitability range (0-1) is divided into two equal intervals: 0.0–0.5 and 0.5–1.0. In each dispersal event, two dispersers will move from a colonized cell if the suitability of that cell exceeds 0.5, and only one disperser will move if the suitability is less than or equal to 0.5”.*

*Interested readers can get adequate information about these processes from Machado-Stredel et al. (2021).*

- The R package “grinnell” is cited after the parameter description. I suggest moving it to the beginning of the paragraph to improve readability.
- *As suggested by the reviewer, the sentence has been shifted up and placed along lines 181-183.*
- The versions or GitHub commit references for both R packages ("grinnell" and "nichevol") are not provided. For reproducibility, please specify the exact versions used. Also, some methodological details are vague, such as the "default settings" used. mentioned without specifying which parameters were set by default. Please clarify the exact parameters to strengthen reproducibility. I recommend providing precise methodological details, possibly in a supplementary table, as done in Rojas-Soto et al. [2024 (doi 10.1111/jbi.14834)].
- As suggested following modifications were done to indicate the version and GitHub references for the packages “grinnell” and “nichevol”

*“The* ***M*** *simulation procedure was performed using the grinnell R package (version 0.0.22; https://github.com/fmachados/grinnell.git) [84]”*

*“Maximum likelihood reconstruction of ancestral niches was performed using the nichevol R package (version 0.1.20; https://github.com/marlonecobos/nichevol.git) [101]”*

*Regarding the parameter settings, as suggested, a separate supplementary file is created as S2 Table, and the following sentence is added along lines 194–195.*

*“Parameterization of the* ***M*** *simulations is detailed in the supporting information (S2 Table)”*

- Results: The geographic and environmental distribution of *Gymnocalycium* (lines 350–355) is currently presented in the Results section, but this information primarily provides context rather than novel findings. I suggest moving this section to the Introduction, where it would serve as a clearer background for the study.
- *As suggested by the reviewer, the following sentence has been removed from the results and placed in the introduction along lines 76–79.*

*“The biogeographic classification of this region comprises three dominions: Chacoan dominion (CD), Paraná dominion (PD), South Brazilian dominion (SBD), and the South American transition zone (SATZ)”*

- Discussion: The study finds that precipitation niches are largely conserved, while temperature niches show more flexibility. The interpretations are interesting, but the underlying drivers of this pattern remain somewhat speculative. As the study does not aim to directly test correlations between root systems and niche evolution patterns, it would be useful to discuss alternative explanations. Can the different rates of niche evolution be driven by other factors?
- *Thanks for this suggestion. A recent publication (Bauk et al., 2025) has linked the precipitation variable with the seed mass of Gymnocalycium species, and suggested that the precipitation variable is significant in the early stages of growth in Gymnocalycium species. This is in support to our observation of the precipitation niche conservatism. Hence, we have added additional sentences in the ending of the last paragraph of the discussion along lines 572-577.*

*“In addition, a recent study revealed that the seed mass of Gymnocalycium species is related to climatic variables, precipitation in particular [142]. Although Gymnocalycium species are distributed in arid environments, their study found that the precipitation variable is important in the early stages of growth in Gymnocalycium species, adding support to the observed phenomenon of conservatism in the precipitation variable”*

- The authors suggests that *Gymnocalycium* species in certain bioregions can be at greater risk due to climate change, by connecting niche conservatism and extinction risk. Since this is a valuable perspective, I recommend discussing potential conservation strategies that could benefit from these findings. Additionally, since the study does not include ecological niche modeling (ENM) under future climate scenarios, I suggest briefly mentioning the importance of integrating ENM projections in future research to confirm species' vulnerability.
- *Thanks for this suggestion. As suggested, the following sentences have been incorporated at the end of the conclusion.*

*“For the Gymnocalycium species distributed mostly in the mountain regions, in situ conservation programs may not serve the long-term goal. It is not possible for these species to simply move up and down the mountains to mitigate the climate change impacts, as land availability and dispersal capabilities are limited. Most of these species utilize specific climatic bands along elevational gradients. Therefore, future research that focuses on predicting the potential distribution of Gymnocalycium species in different periods (e.g., 2040, 2070, and 2100) under different scenarios of greenhouse gas emissions* *will help decide the viability of species-based in situ conservation strategies, utility of ex situ conservation efforts, and possibility of translocating plants from their current habitat to potential distribution areas on a timely basis, considering the climatic impacts.”*

- Conclusion: The phrase "In conclusion" is unnecessary and can be removed. The section already functions as a conclusion without explicitly stating it.
- *Thanks for this suggestion. The phrase ‘In conclusion’ has been deleted.*
- Biases and limitations: The study employs a Binned Ancestral Range (BAR) reconstruction to analyze niche evolution. While the authors present this as a robust approach, it remains relatively new and has been applied in only a limited number of studies. I encourage the authors to provide a more in-depth discussion of its potential limitations. How does BAR compare to other ancestral niche reconstruction methods in terms of potential biases? What are the main uncertainties associated with this method, and how might they affect the study's conclusions?
- *Thanks for this suggestion. As suggested by the reviewer, the following paragraph is added in the beginning of the discussion.*

*“Recent years have witnessed researchers using various methods and tools to reconstruct the ancestral niches [9]. The main shortfall of all such methods stems from the basic fact that it is difficult to estimate the fundamental niche of a species from the presence points alone [9]. Studies that used the summary statistics based on presence points alone pose higher risk of biased sampling, as the occurrence abundance of a species in a particular environment cannot always be interpreted that this suite of environmental conditions are highly favourable for the given species [9]. Because the observed abundance could be due to either biased sampling, or that it may be the most common environment in the accessible area. The BAR method stands out from the methods outlined [9] for two major reasons: the inclusion of the accessible area hypothesis in the analysis and the explicit incorporation of uncertainty. The methods that do not consider these two factors may produce results at the cost of over- or under-estimation of the true niche changes over time across the phylogeny [8]. The BAR method considers ranged response instead of a single value (median values of variables based on realized niche) which is used in more traditional generalized least squares reconstruction (GLS) approaches to estimate ecological niches. Hence, a potential limitation of the BAR method is the lack of an approach for the quantitative estimation of niche evolutionary rates that are comparable to the rates estimated from the conventional single-value reconstructions [9].”*
